# Intermittent theta burst stimulation for negative symptoms in schizophrenia patients with mild cognitive impairment: a randomized controlled trail

**DOI:** 10.3389/fpsyt.2024.1500113

**Published:** 2025-01-03

**Authors:** Jing Li, Xian Mo, Dan Jiang, Xinyu Huang, Xiao Wang, Tingting Xia, Wei Zhang

**Affiliations:** ^1^ Mental Health Center and Psychiatric Laboratory, the State Key Laboratory of Biotherapy, West China Hospital, Sichuan University, Chengdu, China; ^2^ Big Data Center, Sichuan University, Chengdu, China; ^3^ Psychiatry Department, Jinxin Mental Hospital, Chengdu, Sichuan, China; ^4^ West China Biomedical Big Data Center, West China Hospital, Sichuan University, Chengdu, China

**Keywords:** intermittent theta burst stimulation (iTBS), negative symptoms, schizophrenia, dorsomedial prefrontal cortex (DMPFC), functional near-infrared spectroscopy (fNIRS)

## Abstract

**Background:**

This study aims to evaluate the intervention effect of intermittent Theta burst stimulation (iTBS) on bilateral dorsomedial prefrontal cortex (DMPFC) for negative symptoms in schizophrenia using functional near-infrared spectroscopy (fNIRS) to confirm the therapeutic significance of DMPFC in treating negative symptoms and provide new evidence for schizophrenia treatment and research.

**Method:**

Thirty-nine schizophrenia patients with negative symptoms and mild cognitive impairment were randomly divided into a treatment group (n=20) and a control group (n=19). The treatment group received iTBS in bilateral DMPFC. The control group received the sham treatment. Negative symptoms, cognitive function, emotional state, and social function were assessed at pre-treatment, post-treatment, 4-, 8-, and 12-week follow-ups. Brain activation in regions of interest (ROIs) was evaluated through verbal fluency tasks. Changes in scale scores were analyzed by repeated measures ANOVA.

**Result:**

After 20 sessions of iTBS, the Scale for the Assessment of Negative Symptoms (SANS) total and sub-scale scores significantly improved in the treatment group, with statistically significant differences. SANS scores differed significantly between pre- and post-treatment in both groups, with post-treatment scores markedly lower than pre-treatment and better efficacy in the treatment group. However, there was no significant difference in cognitive function, emotional state, and social function. ROIs did not differ significantly between groups before intervention. After treatment, prefrontal cortex activation was significantly higher in the treatment group than in controls, with a statistically significant difference. Regarding functional connectivity, the small-world properties Sigma and Gamma were enhanced.

**Conclusion:**

iTBS on bilateral DMPFC can effectively alleviate negative symptoms and enhance prefrontal cortex activation and the small-world properties in patients of schizophrenia.

## 1 Background

Schizophrenia is a heterogeneous disease with a global incidence rate of nearly 1% and is one of the main causes of disability worldwide, contributing to 12.2% of disability-adjusted life-years globally (1). According to the previous study, the incidence of social function deficits among community Schizophrenia patients is as high as 96.5%, and the unemployment rate is between 70% and 90% (2). Besides typical positive symptoms such as hallucinations and delusions, schizophrenia patients also exhibit different symptoms such as negative symptoms (NS), cognitive impairment (CI), and emotional disorders (3). Currently, the treatment of positive symptoms with medication is relatively satisfactory, but the treatment efficacy for NS and CI is poor, and there is a lack of effective intervention measures. Evidence indicates that NS and CI have a greater negative impact on patients’ quality of life and social functioning than positive symptoms in Schizophrenia (4).

Research has shown that there is a correlation between NS and CI, which are related but separable domains with different functional implications. Their neurobiological basis may partially overlap, and they are both associated with prefrontal lobe dysfunction to some extent (4, 5). Saleh et al.’s research suggests that CI and the severity of NS have a positive correlation. They may be involved in different parts of motivational impairment, have unique contributions to motivational behavior, and collectively lead to motivational impairment in Schizophrenia. Moreover, they may have different impacts on treatment methods (6). Robison et al. indicated that CI in Schizophrenia undermines reward sensitivity because cognitive success is inherently linked to internal motivational drive (7). Xu et al. showed that NS, CI, and reward circuit abnormalities are associated with impaired function within and between prefrontal areas and the basal ganglia, and may reduce spontaneous synchrony between the midbrain and medial and lateral prefrontal cortex (8). Therefore, in the treatment of NS, we cannot ignore the impact of CI. We want to explore whether there are differences in the clinical efficacy of intermittent theta burst stimulation(iTBS) in treating NS in patients with schizophrenia under different cognitive states. Therefore, we divide the severity of cognitive impairment into mild cognitive impairment and moderate to severe cognitive impairment. In this study, we only included Schizophrenia patients with NS and mild cognitive impairment (MCI, defined as a MoCA score between 18-26) (9).

As one of the core symptoms of Schizophrenia, NS has a high incidence rate, long duration, and worsens with disease progression (10, 11). In the past, studies classified NS into a single group and often reported them as a single symptomatology domain. However, increasing evidence suggests that the underlying structure of NS is best related to the five domains identified at the 2005 National Institute of Mental Health Consensus Development Conference: anhedonia, avolition, asociality, blunted affect, and alogia (12, 13). Research results in different domains have great significance for determining pathophysiological mechanisms and targeted therapies for NS. However, there is a relative lack of detailed analyses on the efficacy of treatments in different domains. This study will divide NS into five dimensions based on the Scale for the SANS to explore treatment efficacy.

The prefrontal cortex (PFC) is a key brain region involved in executive function and plays a central role in goal-oriented behavior (14). Frontal dysfunction is often observed in patients with schizophrenia (15). Curtin et al.’s study showed that decreased PFC function and changes in sub-cortical connectivity are correlated with NS and CI in schizophrenia (15). The medial prefrontal cortex (mPFC), as an important part of the PFC, plays a crucial role in many brain functions, such as cognitive regulation, emotional regulation, and motivational control (16). It has been demonstrated in rodent models that neuromodulation of the medial prefrontal cortex affects dopamine signaling in the striatum, subsequently altering reward behavior (17). The dorsomedial prefrontal cortex(DMPFC), as an important part of the mesolimbic dopamine system, plays a significant role in the reward circuit (18). Studies have shown that abnormal reward circuits may lead to NS in schizophrenia, such as anhedonia, social motivation, and voluntary impairments. Zhang et al. found that NS are related to abnormal DMPFC function in schizophrenia patients (19). Stimulating the DMPFC to regulate reward circuits and improve PFC dysfunction may be a potential new therapeutic target for treating NS in schizophrenia.

The treatment of NS has always been a focus and challenge in current clinical work. Effective pharmacological treatments for NS are still rare, and exploring other effective treatment measures is an urgent need. Repetitive transcranial magnetic stimulation (rTMS) is a safe and non-invasive neuromodulation technique widely used in the treatment of mental illnesses (20). Evidence suggests that high-frequency rTMS (HF-rTMS) can improve NS in schizophrenia patients and may be a promising treatment approach (21). However, its therapeutic effects and impact on brain function remain controversial (22). iTBS is a patterned HF-rTMS sequence that can deliver a greater number of pulse stimuli in a shorter time, helping to induce long-term potentiation (LTP) in neurological function, target damaged neural circuits, and activate abnormal network connections (23, 24). This stimulation pattern is closer to the physiological state of neural activity, and the stimulation intensity is weaker than that of traditional HF-rTMS, making it a safe and feasible treatment method in clinical settings (25). Zhao et al.’s study indicated that, compared to traditional HF-rTMS, iTBS can significantly reduce NS in schizophrenia patients (26). A recent randomized controlled study on NS in schizophrenia showed that HF-rTMS stimulation of the DMPFC can improve NS, mainly blunted affect and anhedonia (27). However, Bodén et al.’s study reached the opposite conclusion, indicating that iTBS stimulation of the DMPFC had no significant therapeutic effect on the MAP subgroup of NS in schizophrenia. Nevertheless, their study had certain limitations. Firstly, only the MAP subgroup was included, not all patients with NS, which may have led to inclusion bias. Secondly, the study sample size was relatively small (28). Further research is needed to investigate the clinical efficacy of iTBS stimulation of the DMPFC and its effects on PFC function in patients with NS of schizophrenia.

Functional near-infrared spectroscopy(fNIRS) is a non-invasive optical neuroimaging technique that has been increasingly used in cognitive neuroscience research related to various diseases (29). fNIRS can measure changes in the concentrations of oxygenated hemoglobin (HbO) and deoxygenated hemoglobin (HbR) in the cerebral cortex to explore the activation of different cortical regions (30). Based on the neurovascular coupling phenomenon, fNIRS can effectively evaluate neural activity (31). In brain functional imaging, fNIRS is as effective as fMRI, PET, electroencephalography, and other brain functional imaging technologies (32). It is often used in combination with rTMS to explore clinical efficacy and underlying mechanisms. Verbal fluency task (VFT) is a commonly used cognitive task in fNIRS research, which is related to basic neurocognitive activities such as executive function, working memory, and attention. Currently, VFT is widely used in psychiatric research as a sensitive indicator of cognitive deficits that rely on frontal lobe activation. Numerous fNIRS studies have reported that schizophrenia patients exhibit lower activation of HbO in the PFC during VFT compared to healthy controls, demonstrating abnormal brain activation patterns and low task-related functional connectivity (FC) (33, 34). fNIRS is commonly used to explore changes in PFC activation in patients with various psychiatric disorders before and after transcranial magnetic stimulation (35, 36). However, there is a lack of research on how PFC activation changes in patients with NS of schizophrenia before and after iTBS treatment, and the underlying neural mechanisms remain largely unknown, requiring further exploration.

To explore the efficacy and mechanism of iTBS stimulation of bilateral DMPFC in the treatment of NS in Schizophrenia, and to exclude the influence of CI on the treatment of NS in Schizophrenia, we screened patients with NS of Schizophrenia combined with MCI for intervention and evaluated them using fNIRS. We hypothesize that iTBS stimulation of bilateral DMPFC can increase the activation level of the PFC, promote the improvement of patients’ brain functional connectivity, and ultimately improve NS in schizophrenia.

## 2 Methods

### 2.1 Study design and participants

This study was a single-blind, randomized controlled clinical trial. Thirty-nine patients with negative symptoms of schizophrenia who met the inclusion criteria were recruited from Jinxin Mental Hospital. Post-enrollment, the patients were randomly divided into a treatment group and a control group using a random number table method. The treatment group received 4 weeks of active iTBS treatment on the DMPFC [25.84% of the distance from the nasion to the inion on the midline of the scalp] (36), while the control group received sham treatment. During the treatment process, the evaluators remained unaware of the treatment allocation until the study’s completion and the breaking of the random code. The enrolled patients agreed to maintain their current medication regimen throughout the treatment process. This study was conducted at Chengdu Jinxin Mental Hospital from May 2023 to March 2024. The protocol was approved by the Ethics Committee of West China Hospital of Sichuan University and registered as a clinical trial (registration number: ChiCTR2400079548). All patients participated upon providing written informed consent.

The inclusion criteria for the participants were as follows: 1) Age between 18-59 years; 2) Meeting the diagnostic criteria for schizophrenia in the Diagnostic and Statistical Manual of Mental Disorders, Fifth Edition, diagnosed by a psychiatrist based on MINI Plus v 5.0, with a disease course of at least 1 year; 3) Having received second-generation antipsychotic drugs, including Risperidone, Clozapine, Quetiapine, Olanzapine, and Aripiprazole, with stable dosage in the past 1 month (no specific dosage requirements) and maintaining unchanged dosage throughout the entire treatment and follow-up period; 4) Exhibiting significant negative symptoms: a negative symptom score on the Positive and Negative Symptom Scale (PANSS) of not less than 20, and at least one item on the PANSS negative symptom scale ≥ 4 points; 5) All patients having stable clinical conditions, being able to provide informed consent, and all participants having obtained written informed consent; 6) Combined mild cognitive impairment, with a MoCA score of 18-26 (37).

**Figure 1:**
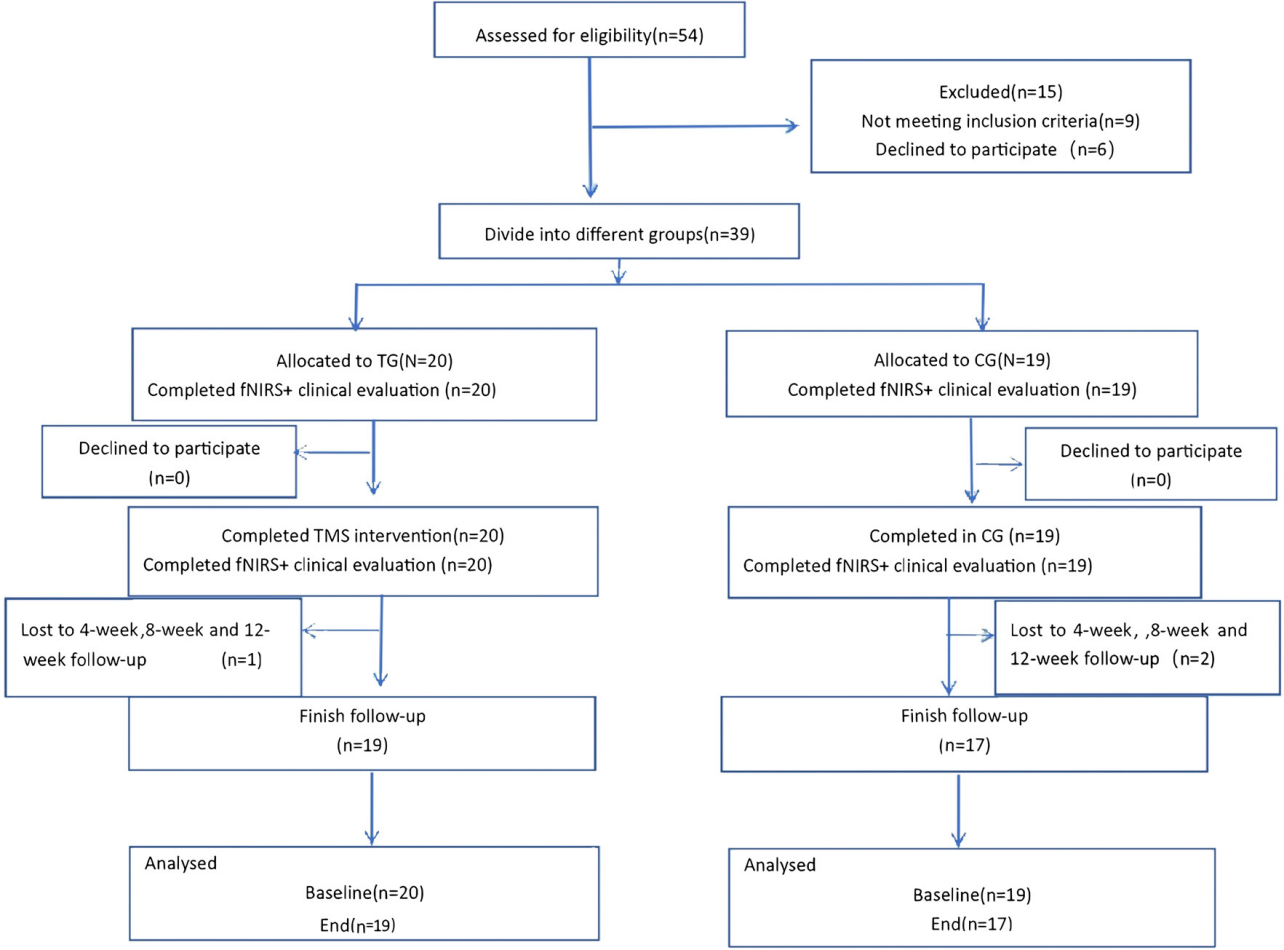
The flow-chart of this study.

The exclusion criteria for this study were as follows: 1) Comorbidity with other brain diseases, such as stroke, epilepsy, or traumatic brain injury; 2) Serious physical conditions, including severe liver, kidney, cardiovascular, and other diseases; 3) History of alcohol and drug abuse, and current substance abuse; 4) Personal or family history of other mental illnesses, including personality disorders and intellectual disabilities; 5) Having metal clips, metal plates, or any other metal objects in the head; 6) Receiving electroconvulsive therapy within 6 months; 7) Pregnancy or breastfeeding; 8) Receiving first-generation antipsychotic drugs and benzodiazepines; 9) Non-cooperation with the assessment and treatment process. The flow-chart of this study is presented in **Figure 1**.

### 2.2 Randomization

Patients were enrolled and randomly divided into two groups using the random number table method. And we generated unique randomization codes for each subject. First, 40 random numbers were generated using a random number generator; then, numbers less than 20 defined group 1, numbers greater than 20 defined group 2. Finally, the intervention method was distributed to the patients based on the random numbers prepared in the consent form. At randomization, patients were randomly allocated (1:1) to the treatment group and control group.

### 2.3 Sample size

According to previous studies, clinical response for patients with schizophrenia was 9% for sham and 54% for active iTBS. Therefore, we assumed that 10% would respond to sham and 54% to active TBS (with alpha = 0.05 and a power of 80%). Based on this, we would need 40 patients (half sham and half active) to detect a clinical treatment effect. With assuming an attrition of 20%, we would need to enroll 50 patients at last.

### 2.4 iTBS program

Participants received iTBS treatment on weekdays, completing a total of 20 treatments. Magnetic stimulation was generated using a CCY-IA magnetic stimulator (Yiruide Medical Co., Ltd., Wuhan, China) and butterfly coils. Before the first TMS treatment, the resting motor threshold (RMT) of the right abductor pollicis brevis muscle was determined as the lowest transcranial magnetic stimulation intensity required to elicit a motor-evoked potential (measured by an EMG system, Yiruide, Wuhan, China) ≥ 50 µV in at least 5 out of 10 trials. In this study, the stimulation target was the DMPFC. The localization method involved having patients wear a coil cap made based on the 10-20 international standard lead system, which needed to be accurately placed on the patient’s nasion, inion, and left and right ears. The stimulation coil consists of two large circular coils forming an obtuse angle, and been placed on the scalp midline at 25.84% of the distance from the nasion to the inion, with the stimulation accomplished by orienting the obtuse angle perpendicular to the scalp, and the handle pointing towards the patient’s lateral side (38). The iTBS treatment parameters were: stimulation intensity of 90% of the resting threshold; each train consisted of 2 seconds of continuous burst stimulation followed by an 8-second rest; each burst consisted of 3 pulse trains at 50 Hz, with bursts repeated every 0.2 seconds at a frequency of 5 Hz, for a total of 1200 pulses. The iTBS stimulation coil was first positioned on the left or right DMPFC, and then switched to the contralateral side for a second identical treatment after one course of pulse stimulation (1200 pulses) was completed. The control group used the sham stimulation coil, which was placed at the same sites as in active treatment and delivered the same stimulation parameters.

To ensure study safety, iTBS intervention safety assessments were conducted once a week. The safety of transcranial magnetic stimulation intervention was assessed through self-reporting, including seizures, headaches, dizziness, nausea, insomnia, facial spasms, etc.

### 2.5 Assessment

#### 2.5.1 Clinical assessments

To evaluate the efficacy of iTBS on DMPFC for NS in schizophrenia patients, we chose the Positive and Negative Symptom Scale (PANSS) (39) to evaluate the psychopathological status of patients before and after treatment; the SANS (40) was used to evaluate the negative symptoms in patients with schizophrenia. The Montreal Cognitive Assessment (MoCA) (37) assessed cognitive function in patients with schizophrenia. The Calgary Depression Scale for Schizophrenia (CDSS) (41) was used to examine the depressive symptoms in schizophrenia patients. The Screening for Deficits in Social Functioning Scale (SDSS) is used to evaluate the social function of patients with schizophrenia. The PANSS scale was evaluated pre-treatment and post-treatment, as well as at the 12-week follow-up. Other scales are evaluated pre-treatment, post-treatment, and at the 4-week, 8-week, and 12-week follow-ups.

#### 2.5.2 fNIRS assessment

**Figure 2:**
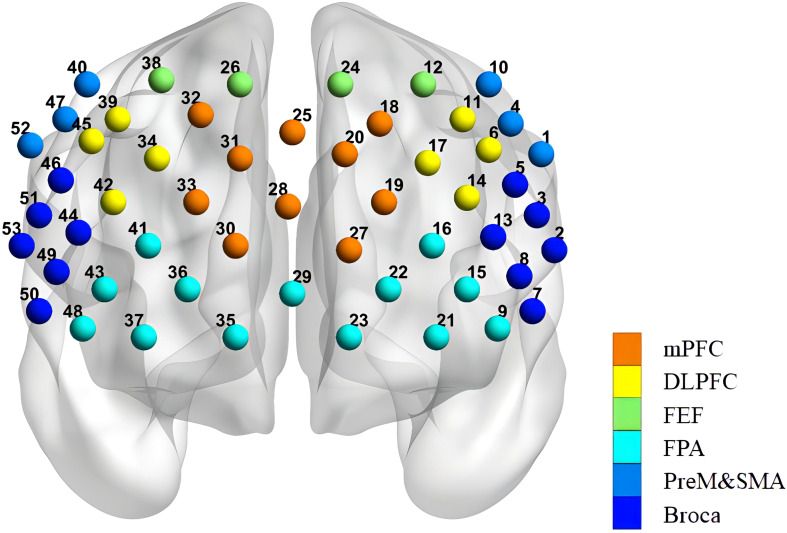
Arrangement of the 53 channels covering the frontal lobe. Including the PreM and SMA (premotor and supplementary motor areas), FEF (frontal eye fields), BROCA (Broca’s area), FPA (frontopolar area), DLPFC (dorsolateral prefrontal cortex), and mPFC (medial prefrontal cortex).

Based on the modified Beer-Lambert law, the fNIRS device (BS-3000, Wuhan Znion Medical Technology Co., Wuhan, China) measures the changes in the concentrations of HbO and HbR in the cerebral cortex using two wavelengths of infrared light (695 and 830 nm). The absorption of infrared light emitted by dual-wavelength laser diodes can distinguish changes in deoxygenated hemoglobin and oxygenated hemoglobin (42). The system consists of 16 light emitters and 16 light detectors, forming 53 channels, with a distance of 3 cm between each emitter and detector. Channels (ch) are defined as the measurement area between the detector and the source-detector pair. The sampling rate is set to 20 Hz. The placement system is made according to the international 10-20 standard lead system, with the lowest probe placed along the Fp1–Fp2 line. The channels are located in the participants’ frontal and temporal lobes. The patients were conducted in a quiet area to reduce unwanted stimuli that could influence the testing; they were asked to sit in a comfortable chair and relax their bodies. And a dedicated data collection specialist observed the patient’s condition throughout the entire process to ensure the accuracy of data collection. The fNIRS was evaluated pre-treatment, post-treatment, and at the 12-week follow-up. The measurement points of the 53 channels and the division of regions of interest (ROI) for NIRS were shown in **Figure 2**.

Resting state: For the resting state test, the subjects were asked to wear the fNIRS detection cap. They were required to keep their eyes open and remain still while looking at the center fixation point (crosshair) displayed in front of them to prevent them from sleeping. The fNIRS measurement began and lasted for eight minutes. When the tests ended, the fixation point disappeared, and the last 30-second rest period began. After the rest period ended, the detection cap was removed, and the detection ended.

**Figure 3:**
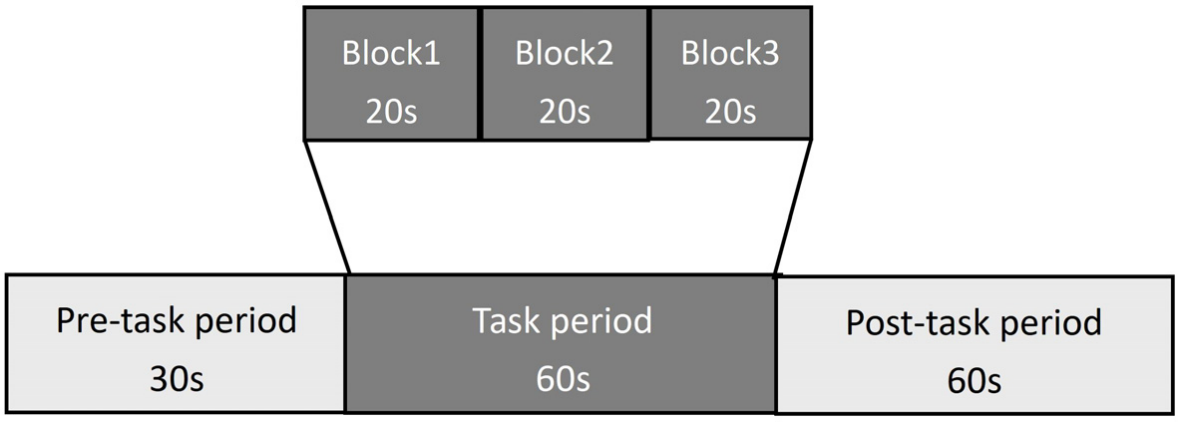
The VFT protocol used for near-infrared spectroscopy. Each trail consisted of a 30s pre-task rest period, a 60s task period subdivided into three 20s blocks, and a 60s post-task rest period.

VFT: In this study, the VFT was developed based on the Chinese population. Each trial included a 30-second pre-task rest period, a 60-second task period, and a 60-second post-task rest period. During the pre-task and post-task rest periods, participants were required to count based on the voice prompts provided by the fNIRS device. The 60-second task period was divided into three consecutive modules, each lasting 20 seconds. In each 20-second module, the subjects needed to name items in a specific category, such as “vegetable names,” “fruit names,” “animal names,” or “furniture names.” Three modules randomly appeared each time, and the subjects were required to name as many items as possible in the relevant category according to the requirements (see **Figure 3**). The testing process was the same for all participants. Before the formal testing, we provided a practice session for all participants to ensure they fully understood the task. During the task period, we monitored the participants’ performance to ensure their full participation in the assessment.

### 2.6 fNIRS data analysis

Data processing was performed using the Homer2 toolbox in the MATLAB environment. Pre-processing procedures were as follows: (1) Converting raw data to optical density (OD); (2) Correcting motion artifacts using a spline interpolation algorithm; (3) Applying a 0.01–0.1 Hz band-pass filter to minimize physiological interference and produce data with the best signal-to-noise ratio, such as blood pressure (Mayer) waves (∼0.1 Hz), respiration (∼0.4 Hz), and heart pulsation (1∼1. 5 Hz); and (4) Converting optical density to relative HbO/HbR concentration through the modified Beer-Lambert law.

HbO has a superior signal-to-noise ratio and can reflect task-related cortical activation more directly than HbR. Therefore, we analyzed the differences in HbO between the two groups of patients before and after treatment. First, we used the HRF function to correct for auto-correlation in the data, and then we used wavelet filtering to filter the data. Finally, the β value was calculated using the general linear model (GLM).

### 2.7 Statistical analysis

Statistical analysis was performed using SPSS 26.0 software (IBM Corp.). Baseline data, such as demographic characteristics and clinical variables, were analyzed using t-tests for continuous variables and chi-square tests for categorical variables. For clinical assessment scales and HbO values before and after treatment, the Shapiro-Wilk normality test was used to evaluate the data distribution of all continuous variables. If the continuous variables followed a normal distribution and had equal variances, they were expressed as mean and standard deviation (mean ± SD). For data that did not satisfy a normal distribution, normalization was achieved through logarithmic transformation. Subsequently, repeated measures analysis of variance (ANOVA) was used for analysis, with changes in PANSS and SANS scores as the main outcome indicators. The within-subject factor was time (baseline, post-treatment, 4-week follow-up, 8-week follow-up, and 12-week follow-up), and the between-subject factor was group (treatment group and control group). For fNIRS-related indicators before and after treatment, if the differences were statistically significant, FDR correction was further used for *post-hoc* comparisons. Estimated mean group differences were reported with 95% confidence intervals, with *P* < 0.05 considered significant and corrected for the false discovery rate (FDR).

## 3 Results

### 3.1 Demographic and clinical characteristics

**Table 1** presents a comprehensive summary of the demographic and baseline clinical characteristics of all study participants. The treatment group comprised 20 participants, with a mean age of 49.15 ± 5.12 years, including 12 males and 8 females. The control group consisted of 19 participants, with a mean age of 45.95 ± 7.97 years, comprising 10 males and 9 females. Statistical analysis revealed no significant differences between the two groups in terms of demographic and clinical characteristics. It is noteworthy that no adverse reactions were reported throughout the treatment process. Detailed information regarding these characteristics is provided in **Table 1**.

**Table 1:** Demographic and clinical characteristics of the study sample.

Characteristics	Treatment Group	Control Group	Group Comparison	
	Mean (SD)/N	Mean (SD)/N	t//x^2^	P
Sex, N (female/male)	8/12	9/10	0.22	0.44
Age (years)	49.15 ± 5.12	45.95 ± 7.97	T=1. 51	0.14
Duration of illness (years)	19.06 ± 8.42	18.21 ± 9.11	T=0.26	0.79
Smoking (cigarette/day)	13.31 ± 6.39	18.88 ± 6.01	T=1. 96	0.07
Education			0.69	0.71
Primary school	7	5		
Middle school	10	12		
College degree or above	3	2		
Marital status			0.68	0.71
Divorce	5	4		
Unmarried	11	9		
Married	4	6		
Antipsychotic drugs			0.90	0.92
Risperidone	14	12		
Clozapine	7	6		
Quetiapine	1	2		
Olanzapine	2	1		
Aripiprazole	2	1		
PANSS-total	77.40 ± 13.65	73.47 ± 10.80	0.99	0.32
PANSS-P	13.05 ± 4.89	13.42 ± 2.85	0.31	0.76
PANSS-N	27.55 ± 4.98	26.37 ± 4.49	0.78	0.44
PANSS-G	36.80 ± 6.75	33.68 ± 6.82	1. 43	0.16
SANS-total	72.70 ± 12.34	72.89 ± 11. 77	0.05	0.96
SANS-affective flattening	20.45 ± 5.25	19.89 ± 4.67	0.35	0.73
SANS- alogia	13.75 ± 3.34	13.95 ± 2.84	0.19	0.84
SANS-avolition	12.40 ± 1. 79	13.11 ± 2.51	1. 01	0.32
SANS-anhedonia	17.15 ± 2.96	17.16 ± 3.37	0.01	0.99
SANS-attentional impairment	8.95 ± 1. 39	8.78 ± 1. 81	0.31	0.76
MoCA	18.95 ± 2.09	19.63 ± 3.65	0.72	0.48
CDSS	1. 55 ± 2.26	0.85 ± 0.89	1. 27	0.21
SDSS	1. 55 ± 1. 43	1. 05 ± 1. 18	1. 18	0.25

### 3.2 Clinical evaluation results after iTBS treatment and follow-up at 4, 8, and 12 weeks

**Figure 4:**
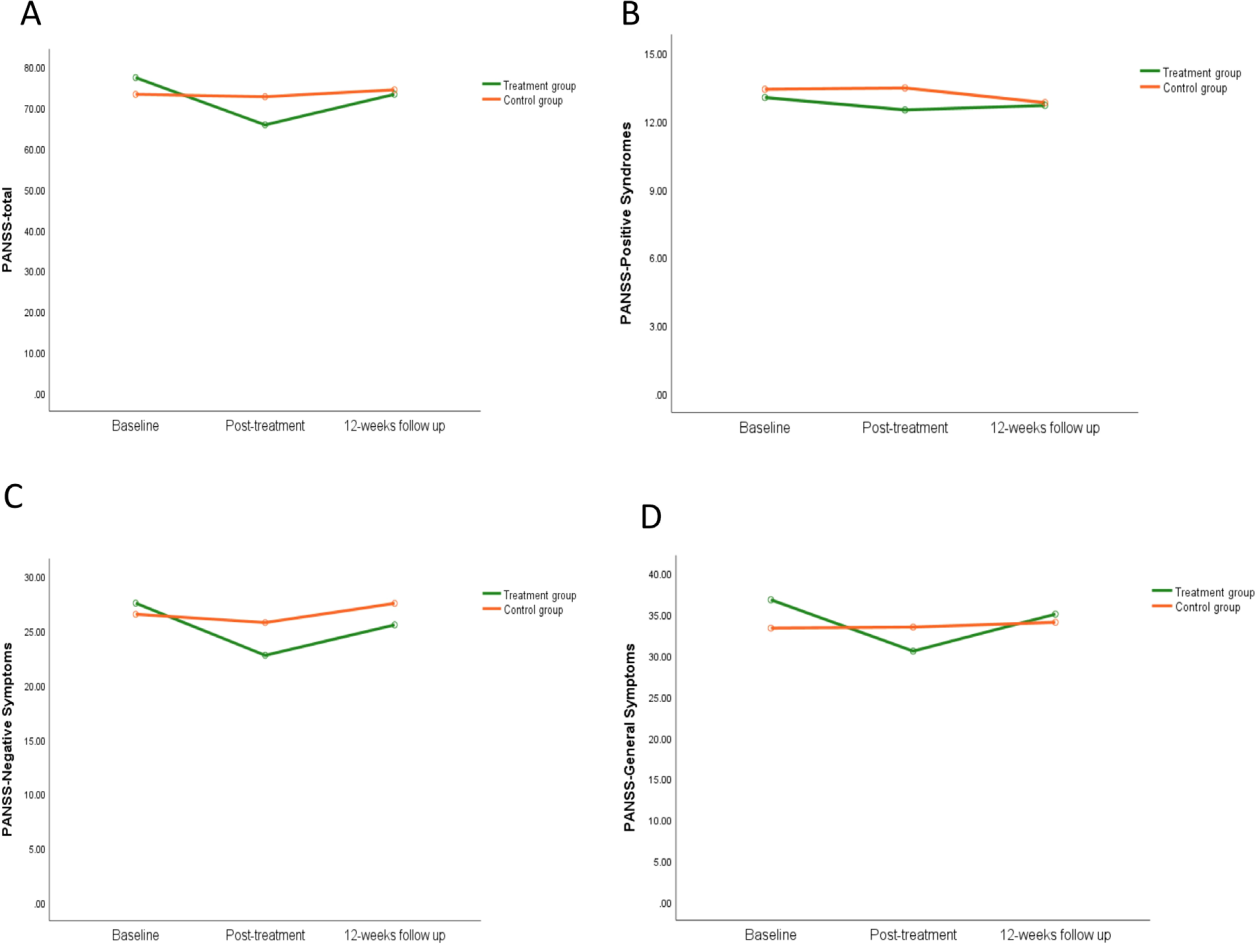
The effect of iTBS on clinical symptoms in schizophrenia patients. Compared to the control group, clinical assessment in schizophrenia patients were completed in the PANSS -total scores **(A)**, PANSS-positive symptoms **(B)**, PANSS-negative symptoms **(C)**, and PANSS-general symptoms **(D)**.

The average PANSS total score stratified by group (treatment group and control group) and time (baseline, post-treatment, and 12-week follow-up) was shown in **Figure 4A**. In the repeated measures ANOVA for PANSS, there were no significant statistical differences in the main effect of group (F=0.12, P=0.74). The main effect of time on PANSS total score (F=18.74, P=0.000) and the interaction between group and time were statistically significant (F=14.08, P=0.000). The PANSS-N and PANSS-G results were similar to the PANSS total results, as shown in **Figures 4C, D**, while PANSS-P showed no statistical differences, as shown in **Figure 4B**. For more information, see **Supplementary Table S1**.

**Figure 5:**
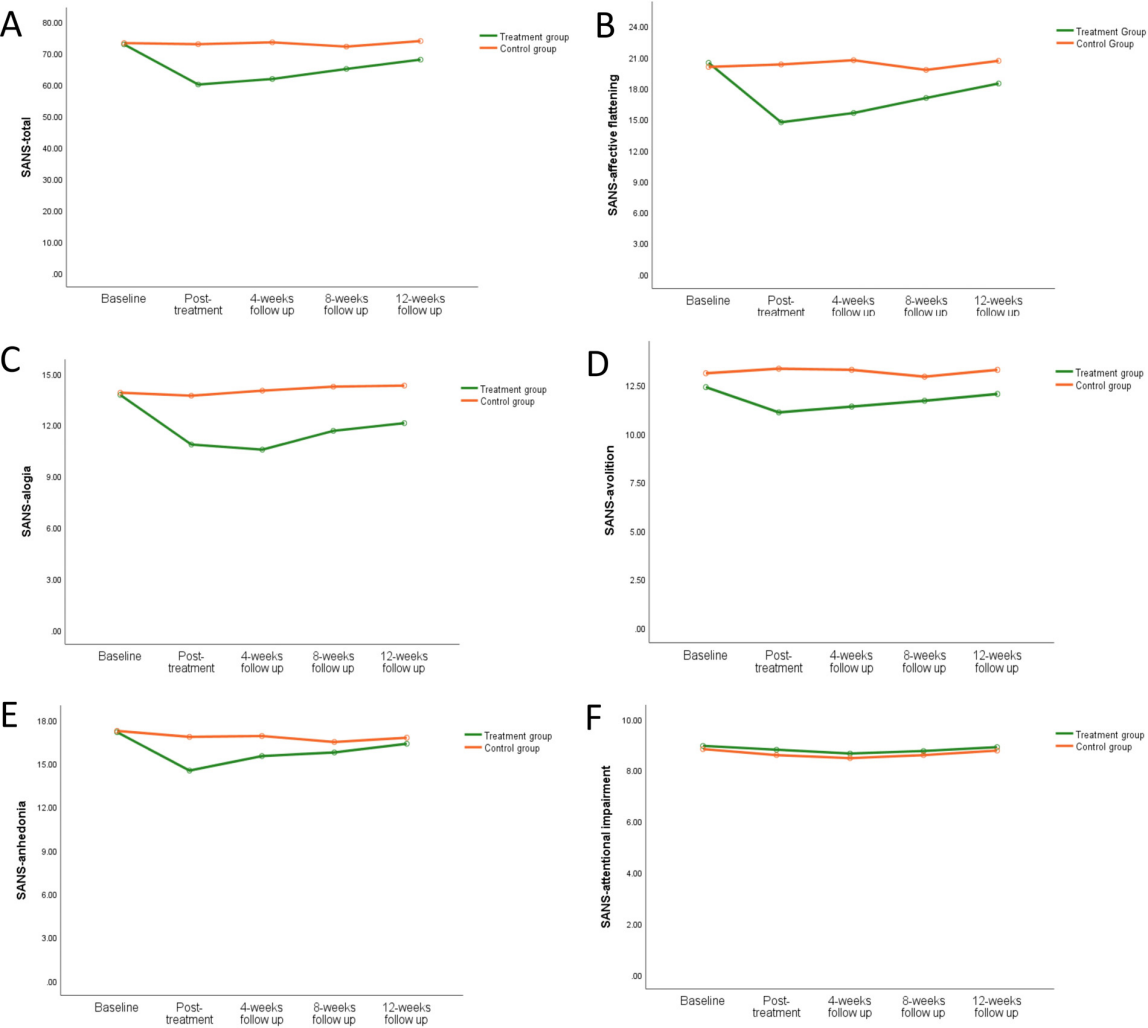
The effect of iTBS on negative symptoms in schizophrenia patients. Compared to the control group, the negative symptoms in schizophrenia patients were improved greatly in the treatment group in SANS total score **(A)**, SANS-affective flattening **(B)**, SANS-alogia **(C)**, SANS-avolition **(D)**, SANS-anhedonia **(E)**, and SANS-attentional impairment **(F)**.

The average SANS total score stratified by group (treatment group and control group) and time (baseline, post-treatment, 4-week follow-up, 8-week follow-up, and 12-week follow-up) was shown in **Figure 5A**. In the repeated measures ANOVA for SANS total score, both the main effect of group (F=6.82, P=0.013) and the main effect of time (F=12.63, P=0.000) were significant, and the interaction between group and time was highly significant (F=11.19, P=0.000). Repeated measures ANOVA was used to analyze the SANS sub-scales. The results for affective flattening and alogia were very similar to the SANS total score, as shown in **Figures 5B, C**. The main effect of group for avolition showed statistical differences (F=5.14, P=0.03), but the main effect of time showed no significant statistical differences (F=2.41, P=0.051), and the interaction between group and time showed statistical differences (F=4.05, P=0.004), as shown in **Figure 5D**. The main effect of group for anhedonia showed no statistical differences (F=1.95, P=0.17), but the main effect of time showed significant statistical differences (F=6.42, P=0.000), and the interaction between group and time showed statistical differences (F=3.87, P=0.005), as shown in **Figure 5E**. There were no significant differences for SANS attentional impairment (**Figure 5F**). For more information, see **Supplementary Table S2**.

As for MoCA, CDSS, and SDSS, no significant group main effect and interaction were found between treatment group and control group (all P > 0.05). For more information, see in **Supplementary Table S3**.

### 3.3 Comparison of pre- and post-measurement HbO indexes in different cerebral regions

Independent sample t-tests and paired sample t-tests were used to test the blood oxygen activities in each cerebral region before and after treatment (cerebral regions: LmPFC, RmPFC, LFEF, RFEF, LDLPFC, RDLPFC, LBroca, RBroca, LFPA, RFPA, LPre & SMA, RPre & SMA; oxygen parameters: HbO).

**Figure 6:**
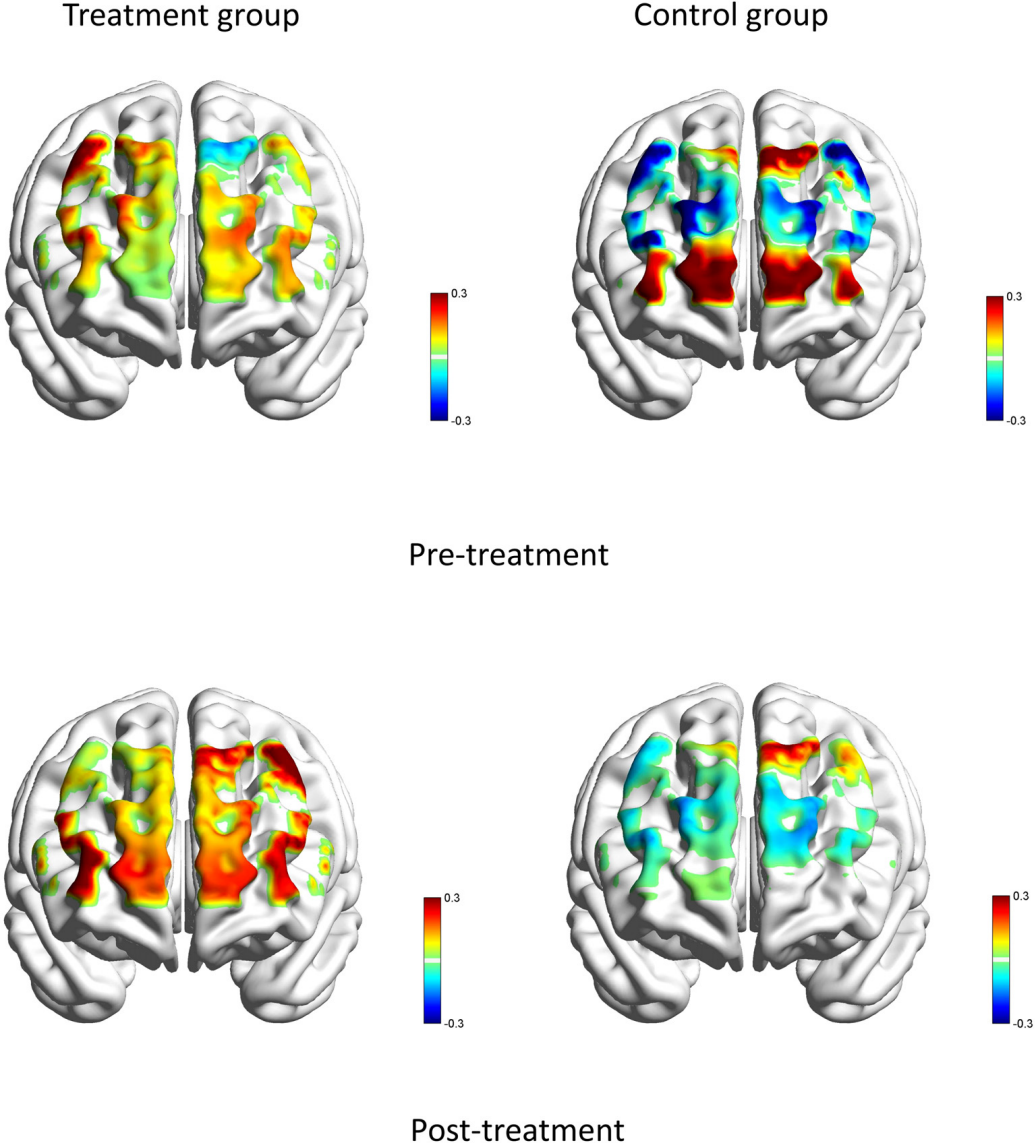
Brain averaged activation maps from changes in HbO for two groups during the VFT pre- and post-treatment.

Before the treatment, there were no significant statistical differences in brain region activation between the two groups. After treatment, during the VFT task, the treatment group showed significantly higher activation in the left premotor and supplementary motor area (T=2.15, P=0.040), left Broca’s area (T=2.77, P=0.010), right Broca’s area (T=3.26, P=0.003), and left dorsolateral prefrontal cortex (T=2.09, P=0.046) compared to the control group (see **Figure 6**). No other significant statistical differences were observed in other brain regions (all P> 0.05).

### 3.4 Analysis of brain network links

**Figure 7:**
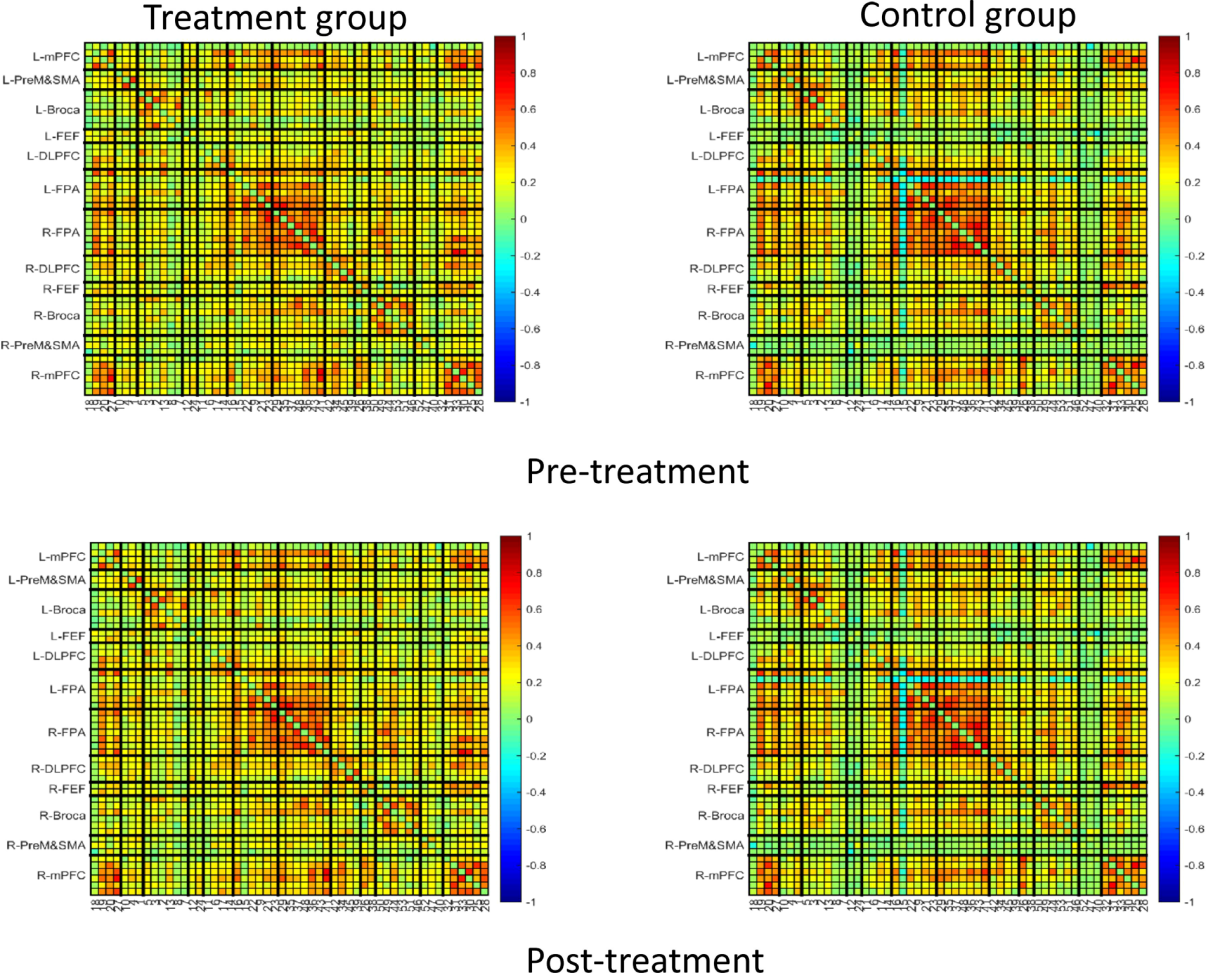
Average function connection metrics in pre-treatment and post-treatment between treatment group and control group.

**Figure 8:**
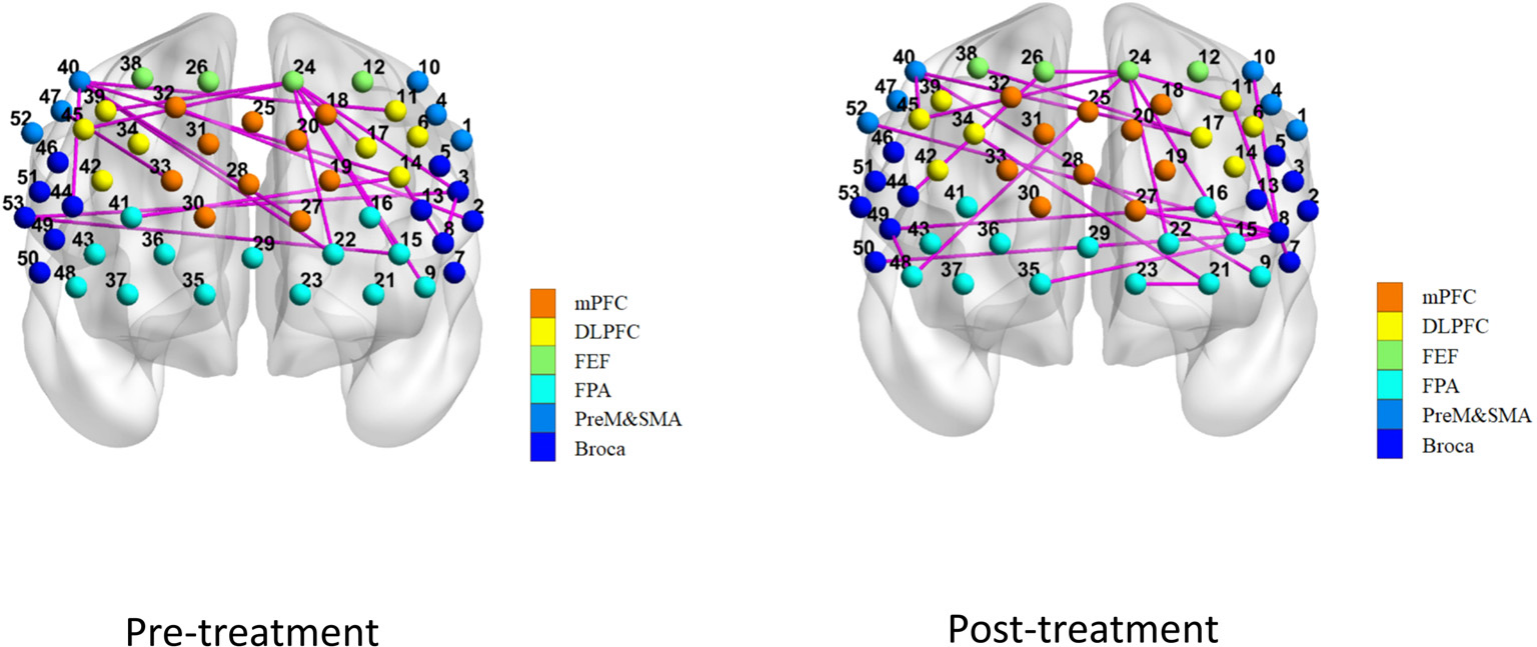
ROI-based function connection metrics without significant inter-group differences.

**Figures 7** and **8** showed the average functional connectivity metrics between two groups pre- and post-treatment, and it was used to evaluate the FC between different brain regions pre- and post-treatment. The results showed that there was no significant difference between the two groups (*P*>0.05).

### 3.5 Analysis of brain network properties

**Figure 9:**
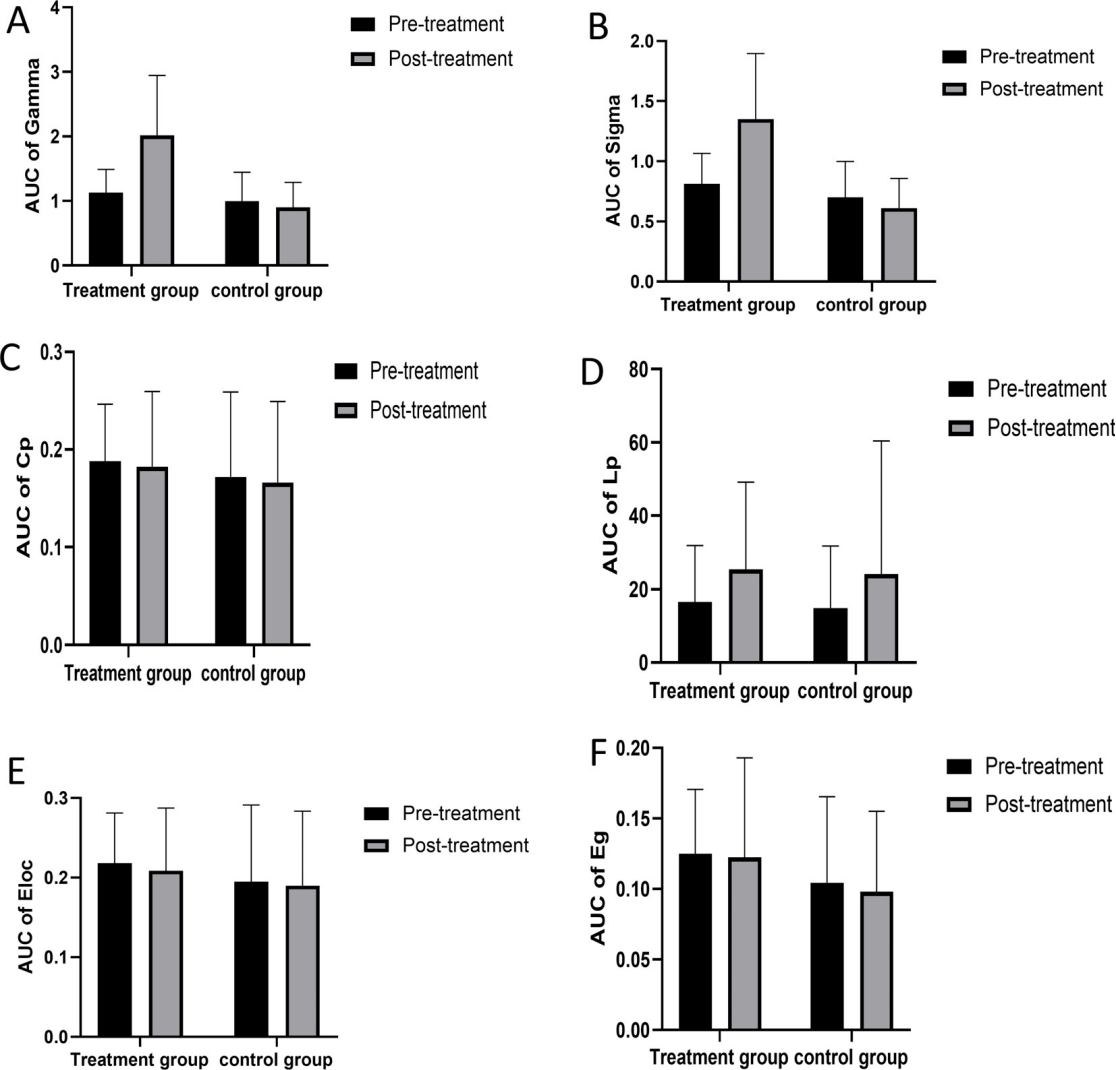
The network properties between treatment group and control group. AUC, area under the curve; Cp, clustering coefficient; Lp, characteristic path length; Eloc, local efficiency; Eg, global efficiency. AUC of Gamma **(A)** AUC of Sigma **(B)** AUC of Cp **(C)** AUC of Lp **(D)** AUC of Eloc **(E)** AUC of Eg **(F)**.

For the network properties, the area under the curve (AUC) of Gamma and Sigma showed significant differences in paired sample t-tests and independent sample t-tests for pre- and post-treatment in the treatment group compared with the control group (see **Figures 9A, B**). After treatment, the small-world sigma and gamma values showed significant improvement in the treatment group (P<0.05) (see **Tables 2**, **3**). The AUC of Cp, Lp, Eloc, and Eg showed no significant differences between the two groups (see **Figures 9C–F**).

**Table 2:** The AUC of Gamma pre- and post-treatment between two groups.

aGamma	Pre-treatment	Post-treatment	T	P
Treatment Group	1. 12 ± 0.36	2.02 ± 0.93	3.758	0.002
Control Group	1. 14 ± 0.24	1. 03 ± 0.17	-1. 311	0.21
T	0.09	3.91		
P	0.93	0.001		

**Table 3:** The AUC of Sigma pre- and post-treatment between two groups.

aSigma	Pre-treatment	Post-treatment	T	P
Treatment Group	0.81 ± 0.25	1. 35 ± 0.54	3.71	0.002
Control Group	0.79 ± 0.13	0.70 ± 0.07	2.64	0.12
T	0.21	4.48		
P	0.83	0.000		

### 3.6 Correlation analysis of negative symptoms (PANSS and SANS) and blood oxygen index changes in each cerebral region

**Figure 10:**
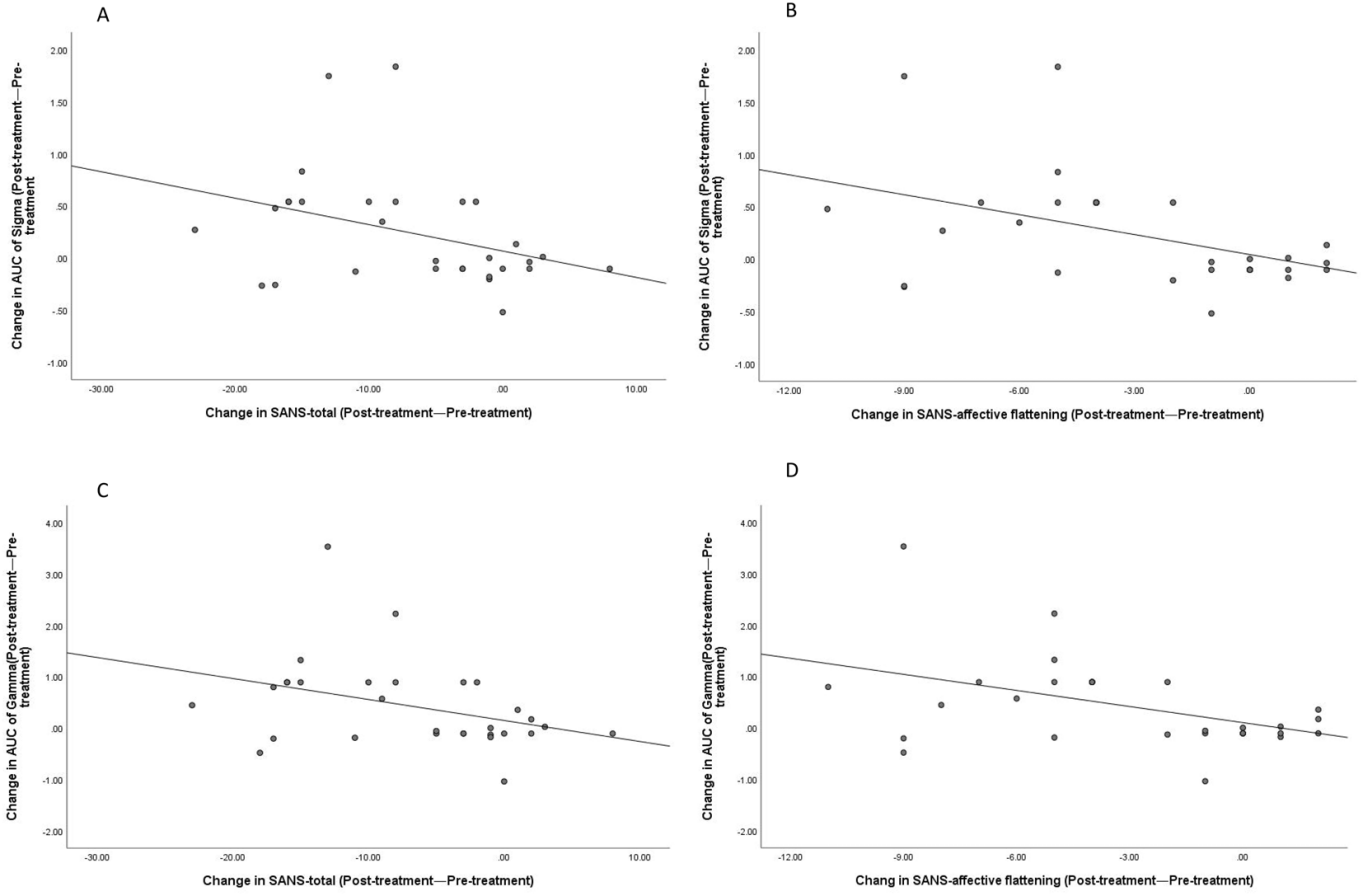
Correlations between changes in SANS-total scores (post-treatment—pre-treatment) and SANS-affective flattening with the changes in AUC of Sigma and Gamma (post-treatment—pre-treatment). Correlation between Sigma and SANS-total **(A)**; Correlation between Sigma and SANS-affective flattening **(B)**; Correlation between Gamma and SANS-total **(C)**; Correlation between Gamma and SANS-affective flattening **(D)**.

Correlation analysis was performed on the changes in PANSS scores and its subscales (post-treatment PANSS score − pre-treatment PANSS score), SANS scores and its subscales (post-treatment SANS score − pre-treatment SANS score), and global property brain connectivity change indicators in the brain connectivity changes (post-treatment global property indicator − pre-treatment global property indicator). The results showed that AUC of Sigma had a negative correlation with the SANS total score (r=-0.367, P=0.046) and a negative correlation with affective flattening (r=-0.445, P=0.014) (see **Figures 10A, B**). AUC of Gamma had a negative correlation with the SANS total score (r=-0.366, P=0.047) and a negative correlation with affective flattening (r=-0.452, P=0.012) (see **Figures 10C, D**). No other significant correlations were found (all *p* > 0.05).

## 4 Discussion

The treatment of NS in schizophrenia has always been a challenge, and drug therapy has shown limited efficacy. Neuroregulatory techniques may be a potential treatment measure. Previous studies have mostly used rTMS to stimulate the DLPFC, but the results have been mixed. In recent years, studies have suggested that the DMPFC may be a promising stimulation target. However, there are still many uncertainties about the brain activation and functional connectivity of schizophrenia patients with NS after iTBS stimulation of the DMPFC. This study combined fNIRS to further explore the changes in brain function. The results showed that after 4 weeks of treatment, the PANSS-N and SANS scores of NS significantly decreased, with statistically significant differences. After iTBS treatment, the NS of schizophrenia patients were effectively relieved, with the most significant improvements being affective flattening and alogia. The therapeutic effects were maintained for at least 8 weeks after treatment, but the symptoms of patients began to slowly rebound 4 weeks after treatment. Avolition showed statistically significant differences between groups, but the differences were not significant across different measurement time points. As time progressed, the differences between the two groups remained statistically significant. However, anhedonia did not show statistically significant differences between groups, but this difference became statistically significant over time. In addition, no adverse events were reported during the treatment process. Stimulation of the bilateral DMPFC by iTBS is a safe and effective treatment strategy, and these results are consistent with the conclusions of Gan et al. (27).

Previous studies had shown that NS had a significant association with the dysfunction of prefrontal-striatal networks. Within this network, key structures, including the ventral and dorsal striatum, medial PFC, orbitofrontal cortex, and DLPFC, may have reduced activation (43). With the relief of NS after treatment, we anticipated that this treatment strategy could cause changes in patients’ brain activation. The results of this study showed that after treatment, the HbO of Broca’s area, PreM & SMA, and DLPFC in patients was significantly higher than in the control group. That is, after treatment, the activation of Broca’s area, PreM & SMA, and DLPFC increased in patients during the VFT task. The increase in blood flow has a positive effect on promoting neuronal synaptic activation (44). Broca’s area is the language center in patients and plays an important role in speech production and language processing. Research has indicated that structural and functional abnormalities in this area are associated with clinical symptoms and CI in schizophrenia patients (45). Specific language-related circuits are impaired, and language-related processing disorders, such as word fluency, constitute the core neuropsychological deficits in schizophrenia. These impairments are believed to be the cause of formal thought disorders in schizophrenia patients and occur early in the onset of the disease (46). Improving Broca’s area activation has positive implications for symptom relief in patients. The results of this study suggest that the increased activation of Broca’s area after iTBS treatment may have positive effects on the improvement of language function and thought disorders in patients. Zhang et al. also pointed out that iTBS treatment can help improve Broca’s area activation in depression patients, and iTBS may become a potential tool for neural remodeling of language and cognitive functions in patients (47). Previous studies have shown that different sub-regions of the mPFC may represent different functions, and the dorsal mPFC and pre-SMA jointly participate in cognitive control processes (48). Sjöberg et al. also pointed out that the Pre-M is not only involved in motor control but is particularly related to higher-level cognitive processes (49). Bender et al. found that the activation of the (pre)motor regions before and around motor execution was significantly reduced in schizophrenia patients (50). In this study, iTBS stimulation of the bilateral DMPFC promoted increased activation of the Pre-M & SMA in patients, which may have positive implications for both motor control and cognitive control. Previous neuroimaging studies have found that NS are associated with decreased activation of the DLPFC (51). Improving DLPFC activation is correlated with the alleviation of NS in patients. Dlabac-de et al. also indicated that the increase in right PFC (including right DLPFC) activation is the first evidence of rTMS treatment for NS in schizophrenia patients: rTMS helps increase the activation of the prefrontal cortex (52). This conclusion is consistent with our research findings.

The results of this study indicate that after iTBS treatment, the small-world properties, Sigma and Gamma, improved to varying degrees in schizophrenia patients. The changes in functional connectivity were negatively correlated with symptom relief, suggesting that the processing ability of local information in the brain may be enhanced after treatment, which could be one of the mechanisms promoting symptom relief in patients. Previous studies have shown that small-world topological structures support the emergence of complex neurodynamics, characterized by achieving the coexistence of functional segregation and integration (53). The brain achieves efficient local and global information transmission with lower energy consumption (54). At the same time, Wang et al.’s research suggests that this dynamic balance helps support the intrinsic needs of different levels of consciousness and cognitive functions, enhances information computing ability, and promotes benefits in other functions (55, 56). Research has shown that schizophrenia is considered a “connectivity disorder” syndrome caused by abnormal neural network formation, and Schizophrenia symptoms are associated with reduced clustering coefficients and changes in local efficiency and small-worldness (57). Zhang et al. found that iTBS treatment can improve brain functional connectivity in depression patients (47). Brady et al. also pointed out that iTBS treatment can promote changes in functional connectivity in schizophrenia patients with NS, and there is a strong relationship between changes in functional connectivity and NS (58).

Motivation impairment and goal-oriented behavioral disorders are considered core features of NS in schizophrenia, particularly closely related to reward deficits (59). Previous studies had shown that an aberrant reward circuit (including aberrant neurotransmitter level, hypo-activation, and hypo-connectivity) was related to NS, with DMPFC playing an important role as a key component of the reward circuit (60, 61). Cho et al.’s research suggests that HF-rTMS stimulation of DMPFC can modulate neurotransmitter levels and circuit integrity in the reward circuit (62). Downar et al.’s study suggests that HF-rTMS stimulation of DMPFC to promote reward circuit integrity may be a reliable neuroimaging predictor of patient functional improvement (63). Therefore, we believe that iTBS stimulation of DMPFC can promote the alleviation of NS in schizophrenia patients by regulating neurotransmitter levels as well as the activity of the PFC and improving the connectivity of reward circuits. In recent years, there have been ongoing studies exploring the DMPFC as a new target for the treatment of various diseases. Struckmann et al. found that iTBS on the DMPFC can promote clinical symptom relief, increase prefrontal oxygenated hemoglobin levels, and improve brain functional connectivity in patients with depression (64). Cheng et al. reached the same conclusion, suggesting that rTMS of the DMPFC can promote symptom improvement and have a positive impact on emotional regulation in patients with depression (65). Fatakdawala et al. indicated that iTBS stimulation of the DMPFC can promote changes in individual dietary behavior, with significantly increased activation of the medial PFC in individuals after treatment (66). Dunlop et al. reported that rTMS treatment targeting the DMPFC can improve symptoms in patients with obsessive-compulsive disorder and alter the functional connectivity between the DMPFC and ventral striatum (67). In research on the treatment of NS in schizophrenia, some scholars have also used the DMPFC as a treatment target (47). Although they pointed to improvements in clinical symptoms, unfortunately, despite these studies producing potential therapeutic improvements, they did not provide an explanation of the mechanisms by which these improvements occur, nor did they demonstrate that the improvements are due to enhancements in brain functional connectivity and activation. This study used bilateral DMPFC as a stimulation target and further explored its impact on brain function in combination with fNIRS. The results indicated that this treatment regimen may have a positive impact on NS and promote PFC activation, but the findings require further validation with a larger sample size in future studies.

This study also has certain limitations. Firstly, the sample size is relatively small, which limits our ability to emphasize differences between the two groups. In the future, we hope to have larger sample size studies to validate our research results. Secondly, our study focuses on a specific subset of people with NS - those with mild cognitive impairment; hence, the generalisability is limited. However, in our other article titled ‘‘Intermittent Theta Burst Stimulation for NS in Schizophrenia Patients with Moderate to Severe Cognitive Impairment: A Randomised Controlled Trail,” the main focus is on patients with NS and moderate to severe cognitive impairment in schizophrenia. Based on the two articles, we believe that CI may affect the treatment of NS in Schizophrenia. This is of great significance for guiding our future research. In the future, we will investigate the therapeutic effect of iTBS combined with cognitive training on schizophrenia and explore effective treatment strategies for NS in Schizophrenia. Thirdly, although fNIRS has its unique advantages, its limitations in terms of insufficient spatial resolution and susceptibility to various factors during the data acquisition process must be objectively recognized. To address the insufficient spatial resolution, a 53-channel device was employed to further improve the accuracy of data acquisition. To mitigate the influence of multiple factors during the data collection process, several measures were implemented. The patients were assessed in a quiet area to reduce unwanted stimuli that could influence the testing, and they were asked to sit in a comfortable chair and relax their body. A dedicated data collection specialist observed the patient’s condition throughout the entire process to ensure the accuracy of data collection. To address the influence of factors such as ambient light, skull thickness, hair density, and head movement, their impact was reduced before data collection by minimizing ambient light and preparing experiments to separate hair follicles during sensor placement. Other factors, such as head movement, can usually be analyzed to reduce interference caused by motion artifacts during later data processing. Finally, we used the scalp localization method determined by Mir-Moghtadaei to locate the site of DMPFC stimulation (38), rather than a more accurate neuronavigation-guided localization approach. However, this localization method has been successfully applied to the treatment of various diseases in previous studies and has demonstrated high consistency with MRI-guided neuronavigation localization methods (67–69).

## 5 Conclusion

The results of this study indicate that iTBS stimulation of the bilateral DMPFC has a positive therapeutic effect on the treatment of NS in schizophrenia, with significant improvements in NS observed in individuals after the intervention. Based on fNIRS, there was a significant statistical difference in brain oxygen levels between the two groups pre- and post-intervention. After treatment, patients in the treatment group exhibited significantly increased blood oxygen levels and activation in the Broca’s area, left premotor cortex (L-PreM), and left dorsolateral prefrontal cortex (L-DLPFC). The treatment helps to enhance functional connectivity between the patients’ brain regions.

## Data Availability

The original contributions presented in the study are included in the article/**Supplementary Material**. Further inquiries can be directed to the corresponding author.
