# Supplementary material for: Intermittent theta burst stimulation for negative symptoms in schizophrenia patients with mild cognitive impairment: a randomized controlled trail
Source: Front Psychiatry. 2025 Jan 3;15:1500113. doi: 10.3389/fpsyt.2024.1500113 (PMC11739303; doi:10.3389/fpsyt.2024.1500113)
Supplement: Supplementary file 1 [file Table1.docx]

Supplementary material

Table1 Results of repeated measures ANOVA for PANSS

|  | Treatment group  (Mean±SD) | Control Group  (Mean±SD) | F  Group | P | F  Time | P | F  time*group | P |
| --- | --- | --- | --- | --- | --- | --- | --- | --- |
| **PANSS-total**  Baseline | 77.40±13.65 | 73.47±10.80 | 0.12 | 0.74 | 18.74 | 0.000 | 14.08 | 0.000 |
| Post-treatment | 65.80±11.71 | 72.84±10.55 |  |  |  |  |  |  |
| 12-weeks follow up | 73.30±12.03 | 74.41±12.61 |  |  |  |  |  |  |
| **PANSS-P**  Baseline | 13.05±4.49 | 13.42±2.85 | 0.20 | 0.66 | 1.23 | 0.29 | 1.06 | 0.35 |
| Post-treatment | 12.50±3.53 | 13.37±2.81 |  |  |  |  |  |  |
| 12-weeks follow up | 12.70±3.87 | 12.82±2.07 |  |  |  |  |  |  |
| **PANSS-N**  Baseline | 27.55±4.98 | 26.37±4.49 | 0.89 | 0.35 | 13.96 | 0.000 | 6.97 | 0.002 |
| Post-treatment | 22.75±4.23 | 25.68±4.15 |  |  |  |  |  |  |
| 12-weeks follow up | 25.55±4.11 | 27.53±5.66 |  |  |  |  |  |  |
| **PANSS-G**  Baseline | 36.80±6.75 | 33.68±6.82 | 0.06 | 0.81 | 13.42 | 0.000 | 12.86 | 0.000 |
| Post-treatment | 30.55±5.81 | 33.79±6.41 |  |  |  |  |  |  |
| 12-weeks follow up | 35.05±5.81 | 34.06±7.54 |  |  |  |  |  |  |

Table2 Results of repeated measures ANOVA for SANS

|  | Treatment Group  (Mean±SD) | Control Group  (Mean±SD) | F  Group | P | F  Time | P | F  time*group | P |
| --- | --- | --- | --- | --- | --- | --- | --- | --- |
| **SANS-total**  Baseline | 72.70±12.34 | 72.89±11.76 | 6.82 | 0.013 | 12.63 | 0.000 | 11.19 | 0.000 |
| Post-treatment | 59.60±9.08 | 72.84±9.89 |  |  |  |  |  |  |
| 4-weeks follow up | 61.70±8.82 | 73.28±10.28 |  |  |  |  |  |  |
| 8-weeks follow up | 64.90±7.34 | 72.00±8.51 |  |  |  |  |  |  |
| 12-weeks follow up | 67.85±8.06 | 73.76±9.28 |  |  |  |  |  |  |
| **SANS-affective flattening**  Baseline | 20.45±5.25 | 19.89±4.67 | 5.83 | 0.021 | 13.06 | 0.000 | 15.24 | 0.000 |
| Post-treatment | 14.35±4.23 | 20.26±3.81 |  |  |  |  |  |  |
| 4-weeks follow up | 15.60±4.21 | 20.67±4.07 |  |  |  |  |  |  |
| 8-weeks follow up | 17.05±3.95 | 19.76±3.86 |  |  |  |  |  |  |
| 12-weeks follow up | 18.45±4.11 | 20.65±3.76 |  |  |  |  |  |  |
| **SANS-alogia**  Baseline | 13.75±3.34 | 13.95±2.84 | 8.68 | 0.006 | 5.57 | 0.000 | 5.23 | 0.001 |
| Post-treatment | 10.85±3.31 | 13.78±2.09 |  |  |  |  |  |  |
| 4-weeks follow up | 10.55±2.70 | 14.06±2.07 |  |  |  |  |  |  |
| 8-weeks follow up | 11.65±2.41 | 14.23±2.46 |  |  |  |  |  |  |
| 12-weeks follow up | 12.10±2.81 | 14.29±2.71 |  |  |  |  |  |  |
| **SANS-avolition** Baseline | 12.40±1.79 | 13.11±2.51 | 5.14 | 0.03 | 2.41 | 0.051 | 4.05 | 0.004 |
| Post-treatment | 11.10±1.68 | 13.37±3.06 |  |  |  |  |  |  |
| 4-weeks follow up | 11.40±1.82 | 13.28±2.35 |  |  |  |  |  |  |
| 8-weeks follow up | 11.70±1.53 | 12.94±2.22 |  |  |  |  |  |  |
| 1. weeks follow up | 12.05±1.50 | 13.29±2.17 |  |  |  |  |  |  |
| **SANS-anhedonia**  Baseline | 17.15±2.96 | 17.19±3.37 | 1.95 | 0.17 | 6.42 | 0.000 | 3.87 | 0.005 |
| Post-treatment | 14.50±1.79 | 16.89±2.73 |  |  |  |  |  |  |
| 4-weeks follow up | 15.50±1.57 | 16.89±2.73 |  |  |  |  |  |  |
| 8-weeks follow up | 15.75±1.97 | 16.47±2.29 |  |  |  |  |  |  |
| 12-weeks follow up | 16.35±1.93 | 16.76±2.36 |  |  |  |  |  |  |
| **SANS-attentional impairment**  Baseline | 8.95±1.39 | 8.78±1.81 | 0.21 | 0.65 | 0.91 | 0.45 | 0.02 | 0.97 |
| Post-treatment | 8.80±1.19 | 8.53±1.31 |  |  |  |  |  |  |
| 4-weeks follow up | 8.65±1.35 | 8.39±1.69 |  |  |  |  |  |  |
| 8-weeks follow up | 8.75±0.91 | 8.56±1.06 |  |  |  |  |  |  |
| 12-weeks follow up | 8.90±0.97 | 8.76±1.15 |  |  |  |  |  |  |

Table3 Results of repeated measures ANOVA for MoCA,CDSS,SDSS

|  | Treatment Group  (Mean±SD) | Control Group  (Mean±SD) | F  Group | P | F  Time | P | F  time*group | P |
| --- | --- | --- | --- | --- | --- | --- | --- | --- |
| **MoCA**  Baseline | 18.95±2.09 | 19.63±3.65 | 0.05 | 0.83 | 1.42 | 0.23 | 0.89 | 0.005 |
| Post-treatment | 20.90±2.31 | 18.89±4.21 |  |  |  |  |  |  |
| 4-weeks follow up | 21.05±3.15 | 17.56±5.07 |  |  |  |  |  |  |
| 8-weeks follow up | 19.65±4.96 | 16.47±5.66 |  |  |  |  |  |  |
| 12-weeks follow up | 20.65±5.57 | 18.38±6.11 |  |  |  |  |  |  |
| **CDSS**  Baseline | 1.55±2.26 | 0.84±0.89 | 0.26 | 0.61 | 0.90 | 0.45 | 1.20 | 0.31 |
| Post-treatment | 1.10±1.74 | 0.84±0.76 |  |  |  |  |  |  |
| 4-weeks follow up | 1.05±1.67 | 0.83±1.04 |  |  |  |  |  |  |
| 8-weeks follow up | 0.90±1.68 | 0.88±0.78 |  |  |  |  |  |  |
| 12-weeks follow up | 0.90±1.71 | 1.00±0.89 |  |  |  |  |  |  |
| **SDSS**  Baseline | 1.55±1.43 | 1.05±1.18 | 0.01 | 0.92 | 0.76 | 0.55 | 0.90 | 0.47 |
| Post-treatment | 1.15±1.39 | 1.05±1.27 |  |  |  |  |  |  |
| 4-weeks follow up | 0.85±0.99 | 0.94±1.26 |  |  |  |  |  |  |
| 8-weeks follow up | 1.15±1.53 | 1.06±1.52 |  |  |  |  |  |  |
| 12-weeks follow up | 0.90±1.48 | 1.38±1.36 |  |  |  |  |  |  |
